# Creation of a Curated Aquatic Toxicology Database: EnviroTox

**DOI:** 10.1002/etc.4382

**Published:** 2019-04-01

**Authors:** Kristin A. Connors, Amy Beasley, Mace G. Barron, Scott E. Belanger, Mark Bonnell, Jessica L. Brill, Dick de Zwart, Aude Kienzler, Jesse Krailler, Ryan Otter, Joshua L. Phillips, Michelle R. Embry

**Affiliations:** ^1^ The Procter & Gamble Company Cincinnati Ohio USA; ^2^ The Dow Chemical Company Midland Michigan USA; ^3^ US Environmental Protection Agency Gulf Breeze Florida; ^4^ Environment and Climate Change Canada Gatineau Ontario Canada; ^5^ Mermayde Groet The Netherlands; ^6^ European Commission Joint Research Centre Ispra Italy; ^7^ Middle Tennessee State University Murfreesboro Tennessee USA; ^8^ Health and Environmental Sciences Institute Washington DC USA

**Keywords:** Database, Aquatic toxicity, Toxicological threshold of concern, Environmental toxicology

## Abstract

Flexible, rapid, and predictive approaches that do not require the use of large numbers of vertebrate test animals are needed because the chemical universe remains largely untested for potential hazards. Development of robust new approach methodologies and nontesting approaches requires the use of existing information via curated, integrated data sets. The ecological threshold of toxicological concern (ecoTTC) represents one such new approach methodology that can predict a conservative de minimis toxicity value for chemicals with little or no information available. For the creation of an ecoTTC tool, a large, diverse environmental data set was developed from multiple sources, with harmonization, characterization, and information quality assessment steps to ensure that the information could be effectively organized and mined. The resulting EnviroTox database contains 91 217 aquatic toxicity records representing 1563 species and 4016 unique Chemical Abstracts Service numbers and is a robust, curated database containing high‐quality aquatic toxicity studies that are traceable to the original information source. Chemical‐specific information is also linked to each record and includes physico‐chemical information, chemical descriptors, and mode of action classifications. Toxicity data are associated with the physico‐chemical data, mode of action classifications, and curated taxonomic information for the organisms tested. The EnviroTox platform also includes 3 analysis tools: a predicted‐no‐effect concentration calculator, an ecoTTC distribution tool, and a chemical toxicity distribution tool. Although the EnviroTox database and tools were originally developed to support ecoTTC analysis and development, they have broader applicability to the field of ecological risk assessment. *Environ Toxicol Chem* 2019;9999:1–12. © 2019 The Authors. Environmental Toxicology and Chemistry published by Wiley Periodicals, Inc. on behalf of SETAC.

## INTRODUCTION

The need for rapid and predictive methods to address ecological hazards of diverse substances is essential because the chemical universe remains largely untested. Flexible approaches that do not require the use of large numbers of vertebrate test animals (fish, amphibians, birds, etc.) are needed to address broad animal welfare concerns as well as optimize resource use. To appropriately develop robust new approach methodologies and nontesting approaches, existing information must be made available via integrated and curated data sets. Increasing regulatory requirements have laid the foundation for the development of more standardized and extensive data sets for a broader range of chemicals. Regulatory programs such as Registration, Evaluation, Authorisation and Restriction of Chemicals (REACH; European Commission 2007); the International Council of Chemical Associations High Production Volume Chemicals Challenge (International Council of Chemical Associations 2018); and Canada's Domestic Substance List (Environment and Climate Change Canada 2018) have helped to create an unprecedented amount of toxicity data along with continuing investigations of hazards of substances to aquatic life.

The threshold for toxicological concern (TTC) is a pragmatic nontesting approach that establishes exposure levels for chemicals below which no appreciable risk to human health or the environment is expected based on a de minimis value for toxicity identified for many chemicals (US Food and Drug Administration 1993; Kroes et al. 2004). The TTC can then be compared to an estimate of the likely exposure to a chemical to complete a screening‐level safety assessment for a given route of exposure or environmental compartment/species of concern. The TTC concept is well established for assessing human safety of indirect food‐contact substances and has been reapplied for a variety of endpoints including carcinogenicity, teratogenicity, and reproductive toxicity. The TTC has benefits for screening‐level risk assessments, including the potential for rapid decision‐making, fully utilizing existing knowledge, reasonable conservativeness for chemicals used in lower volumes, and reduction or elimination of unnecessary animal tests.

An extension to the human safety TTC concept has been initiated for application in environmental situations, termed the “ecological TTC” (ecoTTC; Belanger et al. 2015). The ecoTTC approach utilizes existing ecotoxicological information, derives predicted no‐observed‐effect concentrations (PNECs) for ecological communities, and calculates threshold values expected to have a de minimis probability that effects would be observed for a given group of compounds. The approach enables the prediction of the toxicity of untested chemicals based on structural attribute (category), mode of action, or functional use and may be useful for assessing chemicals at early tiers of the risk‐assessment process (de Wolf et al. 2005). EcoTTCs can provide hazard levels for chemicals that lack quantitative structure–activity relationships (QSARs), guide product development discussions, assist in chemical prioritization/profiling, support read‐across or category justifications, and inform hazard communication labeling.

As with any new approach methodology, the underlying data are crucial for a robust ecoTTC computation, but existing individual databases are not directly suitable to support ecoTTC development. The current effort developed a robust, curated database containing high‐quality aquatic toxicity studies traceable to the original information source. A data curation effort, under the auspices of the Health and Environmental Sciences Institute's Animal Alternatives in Environmental Risk Assessment Committee, was initiated to obtain a large, diverse data set from multiple sources, with harmonization and characterization steps to ensure that the information could be effectively organized and mined. The established EnviroTox database (Health and Environmental Sciences Institute 2019) fully utilizes existing information and associated tools on the EnviroTox platform to allow prioritization of testing for those substances with the greatest environmental hazards.

Ecotoxicity databases act as centralized repositories and should allow easy retrieval of information critical to facilitating the characterization of adverse ecological effects, assessing hazards, and quantifying species‐specific or trophic level–specific risks (Bejarano et al. 2016). These repositories are often tailored to meet the needs of the user community or specific purposes, such as the US Environmental Protection Agency's (USEPA) Interspecies Correlation Estimation application (Raimondo et al. 2016) for extrapolating toxicity between species. Similarly, juvenile fish toxicity data and fish embryo test data for a wide variety of taxa covering a range of chemicals have been compiled to facilitate comparative analyses (Belanger et al. 2013; Busquet et al. 2014). Databases are also developed for more general purposes, such as the USEPA's ECOTOXicology Knowledgebase (US Environmental Protection Agency 2018) as well as the European Chemicals Agency's publicly available REACH submission data (European Chemicals Agency 2018). Other databases house information specific to particular chemical classes (e.g., the USEPA's pesticide ecotoxicity database, pharmaceutical databases) or tests conducted within a particular laboratory (e.g., the Japan Ministry of Economy Trade and Industry database, the US Geological Survey [USGS] Columbia data).

There are multiple challenges to compiling and curating information with the aim of developing a database or library of information. As noted, ecotoxicity information has been available in multiple databases and within various “silos” (e.g., physico‐chemical properties, toxicity, and taxonomy), and careful data collection, verification, and harmonization are required. In addition, many data sets are considered confidential business information and are not readily or easily made available.

Although the EnviroTox database was originally developed to support ecoTTC analysis and development, it has broader applicability to the field of ecological risk assessment. Information can be used to explore patterns of ecotoxicity based on mode of action or assess organism sensitivity patterns within a certain grouping of chemicals, to compare acute–chronic ratios across chemicals and taxa, or determine the relative sensitivity of taxa and toxicity of classes of chemicals. These possibilities are just examples of how robust, comprehensive information could improve the science behind and application to regulatory ecotoxicology (Staveley and Wentsel 2016). The EnviroTox database is publicly available via a web‐based platform that allows for easy searching and direct application to several associated tools (PNEC calculator, ecoTTC tool, and chemical toxicity distribution [CTD] tool) by allowing data selection based on the specific needs of the end user. The present study describes the development, curation, and architecture of the EnviroTox database; summarizes database content; and recommends how these data can be linked, queried, and utilized for assessment purposes with a focus on ecoTTC and related evaluations.

## METHODS

### Ecotoxicology data selection

The Stepwise Information‐Filtering Tool (SIFT) methodology (Beasley et al. 2015) was used to provide a framework for objective data selection and curation, with the final goal of developing a useful and appropriately comprehensive database to support the application of ecoTTC concepts. The SIFT “Step 0” involves defining the scope of the initial “master” data set to which further steps of selection and curation criteria are applied in a stepwise manner. In the present study, Step 0 was defined as a comprehensive, diverse set of ecotoxicological data.

To identify data suitable for calculating PNECs and subsequent ecoTTC or CTD, criteria were established to ensure that the data set had a sufficient number of records and appropriate chemical and taxa diversity. Considerations included multiple chemical classes, modes of action, and functional uses, as well as diversity across trophic grouping. Multiple data sources (public and private sector), geographic regions (global representation), and stakeholder (academia, government, industry) were evaluated when selecting candidate data sources for the master data set.

Sources of ecotoxicological data identified in Table 1 were mined to provide the initial master data set. Information was compiled by associating individual Chemical Abstracts Service (CAS) registry numbers with ecotoxicological data. Each individual data point within a study was considered as a separate entity. The potential to include a data point or study was based on the SIFT methodology (Table 2) where predefined inclusion criteria are used to address relevance, validity, and acceptability of data (Beasley et al. 2015). Effects data were included (i.e., survival, growth, reproduction) that corresponded to known endpoints used in regulatory decision‐making for chemicals identified in standard test guidelines.

**Table 1:** Candidate sources of ecotoxicological data for inclusion in the EnviroTox database as of October 2016

Data source	Description	No. of records/information (SIFT step 3)
ECHA (REACH)	Obtained by query of the REACH data from eChemPortal database of publicly available substance data, submitted to ECHA under the REACH regulations (Organisation for Economic Co‐operation and Development 2018)	2398 records 215 substances 131 species
USEPA ECOTOX	Obtained by query of the USEPA's ECOTOX Knowledgebase, including USEPA‐generated test data and data from the public literature (US Environmental Protection Agency 2018)	68 716 records 1864 substances 955 species
Peer‐reviewed literature	Original data set foundational to species sensitivity distribution work by De Zwart (2002) and colleagues, personal communication to the HESI Technical Committee, containing data and metadata stripped from peer‐reviewed literature	29 903 records 3447 substances 1557 species
AiiDA	Aquatic Impact Indicator Database; contains data sourced from ECHA, ECOTOX, and others. Queried to supplement for data not found in REACH (http://aiida.tools4env.com	2709 records 533 substances 146 species
METI	Summary of aquatic toxicity test results from OECD guideline tests conducted by the Japanese Ministry of the Environment. Some data publicly available via the OECD Toolbox. Also known as the NITE‐CHRIP database (http://www.nite.go.jp/en/chem/chrip/chrip_search/srhInput)	2787 records 464 substances 3 species
FET	Data set of acute aquatic toxicity test results from the OECD validation study to evaluate the reproducibility of the zebrafish embryo test (Belanger et al. 2013; Busquet et al. 2014)	2516 records 229 substances 7 species
USGS Columbia	Columbia Summary data set of acute aquatic toxicity tests conducted by the USGS Columbia Environmental Research Center, including Mayer and Ellersieck (1986; http://www.cerc.usgs.gov)	4053 records 294 substances 66 species
Pharmaceuticals	Summary of acute and chronic aquatic toxicity data for active pharmaceutical ingredients (provided by Sanofi and detailed in Vestel et al. 2016)	334 records 163 substances 3 taxa
ECOSAR training set	Set of aquatic toxicity data used to train the computational QSAR tool ECOSAR (ECOlogical Structure Activity Relationship) developed by the USEPA for hazard estimation; sourced from the help files for the ECOSAR program (US Environmental Protection Agency 2012) (https://www.epa.gov/tsca-screening-tools/ecological-structure-activity-relationships-ecosar-predictive-model)	2311 records 1007 substances 11 taxa
USEPA Pesticide Data	Pesticide Ecotoxicity Database (formerly the Ecological Effects Database); aquatic toxicity data provided by the USEPA's Office of Pesticide Programs Environmental Fate and Effects Division (http://www.epa.gov/pesticides)	516 records 338 substances 15 species
OECD QSAR Toolbox	Queried to supplement aquatic toxicity data from ECOTOX and ECHA; contents include data from ECETOC OASIS and Aquatic Japan Ministry of the Environment (Organisation for Economic Co‐operation and Development 1984)	6178 records 65 substances 60 species

ECHA = European Chemicals Agency; FET = fish embryo test; HESI = Health and Environmental Sciences Institute; METI = Japanese Ministry of the Environment; OECD = Organisation for Economic Co‐operation and Development; QSAR = quantitative structure–activity relationship; REACH = Registration, Evaluation, Authorisation and Restriction of Chemicals; SIFT = Stepwise Information‐Filtering Tool; USEPA = US Environmental Protection Agency; USGS = US Geological Survey.

**Table 2:** SIFT criteria used to ascertain inclusion of ecotoxicological data in the EnviroTox database

Step	Criteria	Specifics	Approximate no. records
0: Purpose	Aquatic toxicity data and metadata	Initial pull of available information from databases listed in Table 1	220 000
1: Relevance	Trophic designations	Fish, amphibian, invertebrate, algae	158 000
2: Validity	CAS	CAS present	132 000
	Required fields	Effect value/units, duration, test statistic, effect measured, source present	
	Qualifiers	Exclude effect values with qualifiers (e.g., <>)	
	Effect	Specific measurement (e.g., EC50)	
3: Acceptability	Duration	≥24 h	122 500
	Test statistic	≥5% and ≤70% effect measure (e.g., IC10, LC50), NOEC, LOEC, MATC	
	Effect	Abundance, biomass, cells, chlorophyll, emergence, filtration rate, gross primary productivity, growth, hatchability, intoxication, mortality, nitrogen fixation, population growth, population reduction, population change, primary production, regeneration, reproduction, shell deposition, teratogenesis	
		Focus is on endpoints of regulatory significance and known use in decision‐making	
4: Additional criteria	CAS, chemical name, SMILES	Harmonized. Database trimmed to only contain validated chemicals	91 000
	Metals	Inorganic compounds were collapsed to a “dummy metal ion” CAS	
	ID of duplicates	Removed records that were full duplicates (e.g., citation, species, test duration, test statistics, measured effect, effect level)	
	Removal of outliers	When a chemical had multiple experimental results, identified and removed outliers within a species and/or trophic level^a^	

^a^ Outliers are defined as 3 orders of magnitude away from the species geometric mean effect value for species tested ≥3 times for any given chemical–species pair; 4 orders of magnitude different from the trophic level geometric mean for a trophic group tested ≥3 times for any given chemical; 3 orders of magnitude from the trophic level geometric mean for a rare species if a trophic group had ≥30 individual entries for a given chemical.

CAS = Chemical Abstracts Service; EC50 = 50% effective concentration; IC10 = 10% inhibitory concentration; LC50 = 50% lethal concentration; LOEC = lowest‐observed‐effect concentration; MATC = maximum acceptable toxicant concentration; NOEC = no‐observed‐effect concentration.

Inclusion criteria, shown in Table 2, were selected based on the SIFT methodology a priori for each successive step, addressing the relevance, validity, and acceptability of the data to the stated end goal. A fourth step was added to encompass a data harmonization process. Criteria were not limited by adherence to only the parameters used in standard guideline testing; however, criteria were influenced by factors including regulatory significance.

### Physico‐chemical information

Several compound identifiers were included in the database, as shown in Table 3. Information on specific chemicals is associated with CAS numbers. The CAS number, chemical name, and Simplified Molecular Input Line Entry Specification (SMILES) were systematically verified. This process involved first running all CAS numbers through the USEPA CompTox Chemistry Dashboard (US Environmental Protection Agency [Link]); if chemicals had a CAS number and chemical name match through this tool, they were considered validated, and the corresponding SMILES was extracted. For those chemicals where there was not a match, the CAS number was run through SciFinder (SciFinder 2007) and checked against several chemical identification tools and databases to determine the name and CAS number; then, the SMILES was extracted. Table 3 summarizes additional information, including chemical properties and ECOSAR compound classification.

**Table 3:** Description of information included in the physico‐chemical file for the EnviroTox database

Information	Description
**Chemical descriptors**	
CAS	Chemical Abstracts Service (CAS) number, no dashes or spaces
Chemical name	Commonly employed chemical name.
SMILES	Unified SMILES (Simplified Molecular Input Line Entry Specification) code associated with the chemical and CAS.
Desalted canonical SMILES	Open Babel (Open Babel 2018) was used to generate desalted and canonicalized SMILES for subsequent modeling and chemical categorization.
Molecular weight	Molecular weight in g/mol; generated from desalted SMILES using EpiSuite DermWin (USEPA 2018b)
Log *K*_ow_	Octanol‐water partition coefficient; unitless; EpiSuite KOWWIN (USEPA 2018b) used to populate Log *K*_ow_ from desalted SMILES. Experimental used if available; modeled if no experimental available
Water Solubility	Solubility of the chemical in pure water (25 °C, 1 atmosphere) in mg/L; EpiSuite WSKOW (USEPA 2018b) used to populate water solubility from desalted canonical SMILES. Experimental used if available; modeled if no experimental available. Effect values that are greater than 5x of the water solubility level were flagged but not removed.
ECOSAR Classification	Assignment of chemical class based on desalted, canonical SMILES input to OECD QSAR Toolbox (https://www.qsartoolbox.org/home). Note that compounds may be assigned to multiple ECOSAR groups depending on types of substitutions.
ECOSAR Classification–collapsed	For chemicals where multiple classifications were generated by ECOSAR, the first reported was used. These categories were further collapsed into 46 more general categories. The complete list of ECOSAR classification collapsed assignments is available as Supplementary Information
USEPA New Chemical Categories	Original categories cited in the document “TSCA New Chemicals Program (NCP)/ Chemical Categories” (USEPA 2010).
**Mode of Action (MOA) Classifications**	
Verhaar	Verhaar classes obtained via OECD QSAR Toolbox (https://www.qsartoolbox.org/home).
TEST	Toxicity Estimation Software Tool (TEST) based on the MOAtox broad assignments as described by Barron et al. (2015).
OASIS	OASIS acute aquatic toxicity MOA obtained via OECD QSAR Toolbox (https://www.qsartoolbox.org/home)
ASTER	ASTER (ASsessment Tool for Evaluating Risk) is a rule‐based expert system and is operated on a proprietary basis by US EPA based on the MOA categories in Russom et al. (1997).
**Chemical Categories**	Determined from SMILES
Halogenated	Contains F, Cl, Br, I
Heavy Metal	Contains a heavy metal (metallic element with a density greater than 5)

OECD = Organisation for Economic Co‐operation and Development; QSAR = quantitative structure–activity relationship; USEPA = US Environmental Protection Agency.

### Taxa descriptions

Taxonomic information was collated for all species as summarized in Table 4. Current taxonomic status was harmonized as of October 2016. It is recognized that some taxa designations may be somewhat arbitrary. Common authoritative taxonomic websites including

**Table 4:** Information included in the standalone (noninteractive) species file for taxa found in the EnviroTox database

Latin name	Linnaean genus and species name
Trophic level	Algae, invertebrate, fish, amphibian, macrophyte, fungi
Taxonomic kingdom	Consensus‐based designation
Taxonomic phylum or division	Phylum (animal) or division (plant)
Taxonomic subphylum	Not always available
Taxonomic superclass	Not always available
Taxonomic class	Taxonomic class
Taxonomic order	Taxonomic order
Taxonomic family	Taxonomic family

National Center for Biotechnology Information database, AlgaeBase database, the World Register of Marine Species, and Fishbase were consulted to derive database classifications. Numerous taxa were harmonized to modern taxonomic nomenclature. All species identified in the database were also assigned to a freshwater or saltwater habitat classification. For estuarine or facultatively freshwater to saltwater species, the primary habitat in which they are known or were tested was used to assign the habitat classification.

Ecotoxicological data were not included in the database if tests were performed on mixed communities of organisms (i.e., more than one taxon) or on organisms only described at higher taxonomic levels above the genus (i.e., family or higher). Field‐collected organisms from a single site or apparent population most likely represent a single taxon and were included in the database when tested in traditional laboratory environments (this commonly occurs for many taxa that lack well‐defined propagation techniques under laboratory conditions). At times, field‐collected organisms may also not be sufficiently large to improve taxonomic identification below the level of genus (e.g., early instars of insects). Note that the tests at levels of taxonomic resolution above the genus are identified in the database. Studies that only included taxonomic identity at the level of family and higher were excluded from further analyses and queries of the database using the SIFT process (Beasley et al. 2015).

### Toxicity endpoints

**Figure 1:**
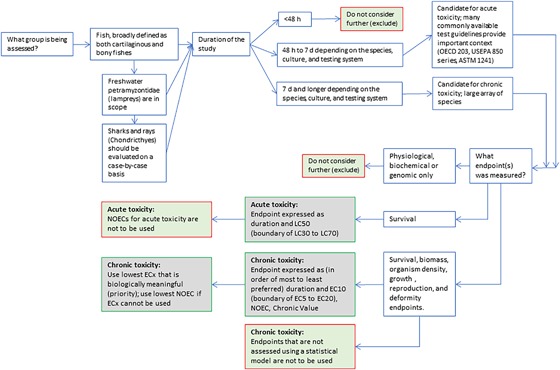
Acute and chronic designations for fish tests in the EnviroTox database. ASTM = ASTM International; EC*x* = *x*% effective concentration; LC50 = 50% lethal concentration; NOEC = no‐observed‐effect concentration; OECD = Organisation for Economic Co‐operation and Development; USEPA = US Environmental Protection Agency.

Decisions on what endpoints to include and how to ascertain if the study was an acute test or chronic test has a significant impact on the potential applications of the data. Endpoints for ecotoxicity studies were evaluated for their utility and previous use in regulatory evaluations of ecotoxicity data (Hall et al. 2017; Moermond et al. 2017; Ruden et al. 2017). Further, the endpoint was then associated with appropriate statistical evaluations to arrive at a conclusion of acute or chronic toxicity or unassignable. As an illustrative example, a study on the ecotoxicity of a chemical to *Daphnia magna* (an accepted cladoceran) was performed over 17 d, and a biomarker response was measured and identified as a no‐observed‐effect concentration. Although the species, duration, and statistic may be appropriate for a “chronic” interpretation, the biomarker response is not presently used in any regulatory framework for environmental risk assessment so would be excluded from the EnviroTox database. Acute and chronic designations were assigned via decision logics for each taxonomic group based on endpoint, species, and statistic. Figure 1 is an example detailing the logic used to classify fish studies as acute or chronic. Additional figures for photosynthetic microbial species (S1), nonphotosynthetic microinvertebrates (S2), macroinvertebrates (S3), amphibians (S4), and macrophytes (S5) are included in the Supplemental Data.

### Mode of action assignments

To allow grouping of chemicals for computational analyses, 4 mode of action classification assignment schemes (Verhaar, TEST, OASIS, and ASTER) were applied to each chemical. This expanded on previous work (Kienzler et al. 2017) on an earlier version of the database. The specific mode of action assignments obtained from each of the 4 schemes are included in the database.

### Additional data curation

#### Metals

The toxicity of some metal‐containing compounds can be driven by the presence of the freely dissolved metal ion. Consistent with the US derivation of water quality criteria for aquatic life and international screening values (Barron and Wharton 2010), specific divalent metal–containing compounds were grouped by metal ion. Inorganic compounds were assigned to a “dummy metal ion CAS” if 1) the metal ion could dissociate (e.g., acetate, lactate), or 2) the toxicity of the metal‐containing compound would be driven by the metal ion. A metal‐containing compound was not assigned to a dummy metal CAS if it was caustic or highly reactive, if the metal was associated with ammonia or hydroxides, or if more than one metal was present in the compound. A total of 140 compounds in the database were assigned to one of 24 different dummy metal ion CAS. The original CAS for the metal‐containing compound and dummy metal ion CAS are both provided in the database.

#### Salts

Chemical compounds were excluded from the database if the desalted canonicalized SMILES resulted in the individual hydroxide (OH^–^), chloride (Cl^–^), ammonium (NH^4+^), or amino cation (NH^2+^). The corresponding effects data for excluded compounds were removed from the database because of uncertainty regarding the moiety that would produce the toxicological effect.

#### Solubility

Effect values ≥5‐fold over the tested chemical solubility were flagged as suspect entries within the database but were not removed. This functionality allows the end‐user to potentially remove these values if desired for specific user‐directed analyses.

#### Effects values

Database entries were evaluated to identify and remove individual tests or assays that may be outliers compared to other tests on the same chemical. It is recognized that outliers may exist in this database for various reasons, including unit entry errors and differences between nominal and measured test concentrations in the original information source. For species that were tested at least 3 times for any given chemical–species pair, an outlier was defined as being at least 3 orders of magnitude away from the species geometric mean. For a trophic group that has been tested at least 3 times for any given chemical, an outlier was defined as being at least 4 orders of magnitude different from the trophic‐level geometric mean. If a trophic group contained at least 30 individual entries for a given chemical and contained a rare species (e.g., a species with only 1 or 2 entries in the database) that shows an effect level at least 3 orders of magnitude from the trophic‐level geometric mean, it was defined as an outlier.

### Database system

The master data set and correlated metadata were compiled into an Access database to allow linking of individual flat data tables by key data fields (e.g., CAS–CAS) to create a semirelational database which could then be manipulated to harmonize or query data. Ecotoxicological test data and correlated metadata (e.g., physico‐chemical properties, taxonomic information) were maintained in standalone tables linked by key data fields (e.g., Latin name–Latin name for species of test organism). Original source data were maintained for context because stepwise filtering and inter‐ and intratable harmonization could potentially permanently remove large sections of data. Whenever possible, data fields were represented only in one table for consistency and efficiency. Relationships were relied on to view or analyze collections of data and metadata. For example, trophic‐level designations were housed only in the “taxonomy” table but could be linked to the “working all data” table using the relational linkage of the Latin name of the test organism used in the study result.

Query systems were developed to mine data for analysis and combine source data for harmonization. As an example of combining original ecotoxicological test data tables for inclusion into the master data set, 10 key data elements were linked (e.g., CAS, chemical name, test organism, test duration) in a union query that returned one set containing only the selected elements. Intratable overlap was identified with query systems using common data elements that might help to quickly identify large sections (e.g., the title of a peer‐reviewed source) or common elements (e.g., combinations of test organism, duration, and endpoint that fit certain guideline tests). When significant overlap was identified, the source was considered to determine which data were eventually included. Duplication was encountered at the intertable level, requiring queries to identify and remove duplicates. The entire database was subjected to duplicate analysis using an R‐script, which flagged studies if they possessed exact matches of key study identifiers (chemical, effect concentrations, study duration, citation, etc.). Some duplication was identified and removed manually because fields such as “source” often misidentified or missed duplicate data because of the complexity of the contents. Some duplication attributable to variations in citation reporting may exist. Further database curation and data exploration were conducted in R (an integrated suite of software facilities for data manipulation, calculation, and graphical display [R Development Core Team 2013]). All information excluded from the database per these points were archived.

## RESULTS AND DISCUSSION

### Database structure

Thirteen original sources of ecotoxicological data were obtained and combined to yield a master data set of approximately 220 000 records. Once initial screens for overlap and duplication were completed, one source was eliminated (USGS Columbia, subsumed in the queried data from ECOTOX) and 2 smaller sets were rolled up into a larger combined table, leaving 10 flat tables to be linked by unique CAS. Once SIFTed, these were combined into the “working” data set of key fields (CAS, SMILES, chemical name, Latin name, effect value, unit, duration, test type, test statistic, effect, and source). Metadata were linked to working data based on the flat file content (e.g., taxonomy to working by Latin name of the test organism). All files were linkable by unique CAS.

### Database summary

**Figure 2:**
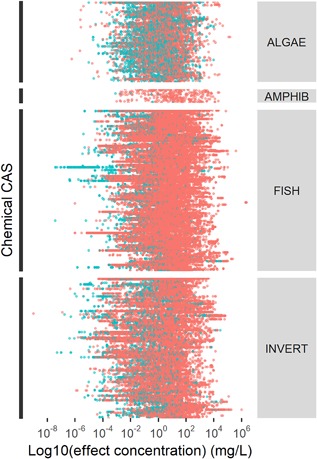
Summary of toxicity values in the EnviroTox database. Effect concentrations are grouped by their trophic level (algae, amphibian, fish, invertebrates) and colored based on the experimental duration (acute = red; chronic = blue). CAS = Chemical Abstracts Service.

The EnviroTox database includes 91 217 acute or chronic study records representing 1563 species and 4016 unique chemical CAS numbers. Acute toxicity data predominate, with most data originating from acute fish or invertebrate studies (Table 5). Figure 2 shows the range of acute and chronic toxicity values across different trophic levels. Effect values span approximately 15 orders of magnitude overall. Individual trophic groups have effect values ranging from 10 orders of magnitude (amphibians) to 15 orders of magnitude (invertebrates); this range of effect concentrations represents a combination of species.

**Table 5:** Summary of EnviroTox database acute and chronic data

	No. of species			No. of entries	
	Total	Acute tests	Chronic tests	Acute tests	Chronic tests
Algae	196	191	89	6376	3998
Amphibian	37	37	5	375	10
Invertebrate	872	854	111	21 565	4066
Fish	458	455	79	50 689	4138

#### Effects information

The database contains 738 chemicals that have acute toxicity data for algae, invertebrate, and fish species (Figure 2); and these data can be used to compare the relative sensitivity among trophic groups. Algae was the most sensitive trophic group, followed by invertebrates and fish (48.8, 28.5, and 22.8%, respectively). Previous research (Hutchinson et al. 2003; Jeram et al. 2005) reported similar results, with algae and invertebrates being more sensitive than vertebrate trophic groups.

#### Species representation

**Figure 3:**
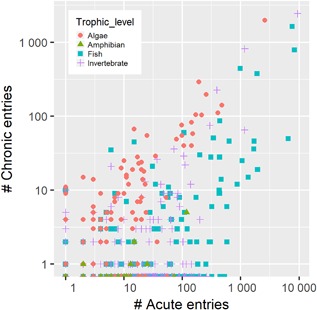
Number of data points for each species in the EnviroTox database. The database contains a total of 196 algae, 37 amphibian, 458 fish, and 872 invertebrate species.

Studies contained within the EnviroTox database are dominated by relatively few individual species (Figure 3). More than 68% of the fish data are derived from 11 species. The most data‐rich fish species have the following total number of database entries: *Pimephales promelas* (9267), *Oncorhynchus mykiss* (9112), *Lepomis macrochirus* (6579), *Oryzias latipes* (2283), *Ictalurus punctatus* (1973), *Cyprinus carpio* (1741), *Poecilia reticulata* (1712), *Danio rerio* (1428), *Carassius auratus* (1366), *Gambusia affinis* (1081), and *Oncorhynchus kisutch* (882). Databases generally have an abundance of fish data (e.g., numerically overrepresented) because of their high societal interest, although they are the least taxonomically diverse group (Mora et al. 2011). There are records for 929 invertebrate species in the database, though they are dominated by results from 4 freshwater species: the crustacean zooplankton *D. magna* (11 898), *Ceriodaphnia dubia* (1990), *Daphnia pulex* (1229), and the rotifer *Brachionus calyciflorus* (617). Collectively, these data represent 61% of all invertebrate data in the EnviroTox database. In contrast, 44% of the algal data are derived from a single species, *Pseudokirchneriella subcapitata* (4593 entries). Other data‐rich algal species include *Desmodesmus subspicatus* (618), *Chlorella pyrenoidosa* (517), *Anabaena flos‐aquae* (486), and *Chlorella vulgaris* (453). Generally, algal records are underrepresented in EnviroTox and other databases, which reveals an important issue because algae are frequently the most sensitive taxon (Rawlings et al. 2019). The database contains very little amphibian data by comparison. There are 385 amphibian data points, with the majority of the data from studies conducted with *Xenopus laevis* (124) or *Rana limnocharis* (72). Although the EnviroTox database is dominated by experimental studies conducted with well‐known, standard regulatory species, species diversity in the candidate data for inclusion into the database was high, with estimates of 10 000 to 15 000 species covered in the source databases. In contrast, this may be only 0.2% of global species diversity (Mora et al. 2011).

#### Chemical representation

A diversity of physico‐chemical properties and mode of action classifications have been included in the database to facilitate data interpretation and/or the development of more localized statistical distributions or other computational analyses. When appropriate, chemical properties for each of the approximately 4000 chemicals were modeled using EPISuite. Model output for molecular weight, log octanol–water partition coefficient (*K*_OW_), and water solubility were collected. Chemicals had a wide range of (desalted–counter‐ions removed) molecular weight from 6.94 to 1701 (mean = 245.9, median = 240.3) g/mol. Log *K*_OW_ values ranged from −9.14 to 24.77 (mean = 2.91, median = 2.92). Water solubility values included a combination of measured and modeled values ranging from 1.78 × 10^−23^ to 6.54 × 10^6^ (mean = 94 844, median = 81) mg/L. A total of 5761 studies (6.3% of the database) reported an effect value that was more than 5 times greater than the water solubility limit. These values were primarily derived from acute toxicity tests and include over 716 different CAS numbers. A flag has been added to the database to highlight these experimental values.

**Figure 4:**
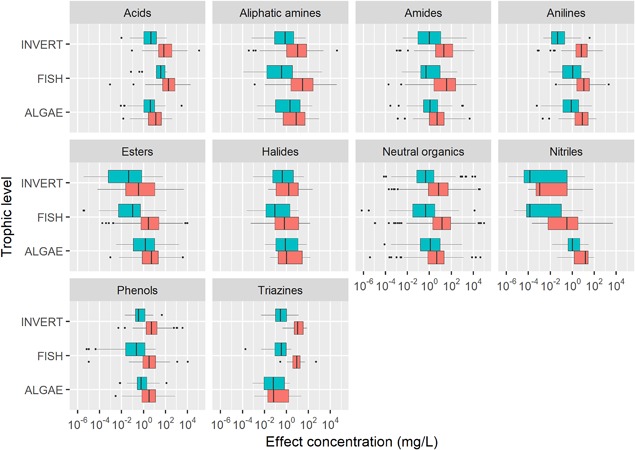
Chronic (blue) and acute (red) toxicity values for the 10 most common ECOSAR classes, separated by trophic level. The box represents the 25th, 50th, and 75th quartiles. Outliers were identified as toxicity values >1.5 times the interquartile range and are plotted as dots. Additional details (number of substances and species) on this figure are included in the Supplemental Data.

More than 25% of the chemicals in the database are classified as esters by ECOSAR based on the collapsed categories (Supplemental Data, Table S1). Other prominent ECOSAR classes include neutral organics (19.4%), phenols (12.3%), amides (4.4%), and aliphatic amines (4.3%). The acute and chronic toxicity values of the 10 most common ECOSAR classes are shown in Figure 4.

#### Mode of action representation

**Figure 5:**
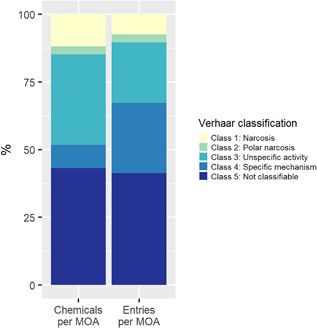
Chemical and data coverage within the Verhaar mode of action scheme with all acute data in the EnviroTox database. MOA = mode of action.

The mode of action for each chemical was modeled using TEST, OASIS, ASTER, and Verhaar (Organisation for Economic Co‐operation and Development [OECD] QSAR Toolbox) classification tools. Chemicals that were outside the domain of applicability of these models (e.g., metals) were labeled as not classifiable or unclassified. A significant portion of the EnviroTox database is not classifiable because of either inability of the 4 schemes to assign an mode of action or lack of consensus among the classification schemes. For example, 44% of the chemicals in the EnviroTox database were not classifiable by the Verhaar scheme, which addresses acute toxicity mode of action (Figure 5). This corresponds to 42% of the EnviroTox effects data. The database contains approximately 1300 Verhaar class 3 (unspecific reactivity) chemicals, corresponding to 33% of the EnviroTox database chemicals. However, these chemicals are relatively data‐poor and represent 24% of the effects data. By contrast, the Verhaar class 4 specific‐acting compounds are data‐rich, comprising only 8% of the chemical space in the database but 25% of all entries.

**Figure 6:**
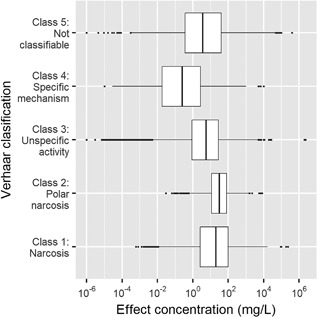
Distribution of acute fish effects, by Verhaar mode of action.

Verhaar mode of action classifications can also be explored by trophic group. We examined the range of acute fish toxicity values across Verhaar classifications (Figure 6). Verhaar class 1 and class 2 chemicals have comparable toxicity. However, as expected, class 4 specific‐acting chemicals are generally more toxic with lower acute toxicity values. The range of observed effect concentrations spans multiple orders of magnitude for all Verhaar classifications. The lack of specificity between these mode of action classes may be an important consideration in the exploration and development of distributions.

#### Data set construction challenges

The construction of any new database must address multiple challenges. The initial development can be driven by an assessment of whether existing databases, information, and tools are fit for the purpose intended. In the case of the EnviroTox database, existing individual databases were not structured in a way that allowed direct ecoTTC derivation and related computational analyses. The construction of the EnviroTox database, with suitable size and complexity, highlighted several overarching issues with implications for future use of the database.

#### Accessibility of data

Although large compilations of data (e.g., ECOTOX, European Chemicals Agency) are theoretically available to the public for investigation, the related query mechanisms are difficult to employ unless the user is highly proficient. Smaller data collections often do not have sophisticated query mechanisms capable of advanced data search and selection. Simply querying a large ecotoxicological database is difficult and may net a data set that still requires significant curation after collection.

Data that are privately held (e.g., industry‐generated test data in compliance with REACH requirements for chemical registration) may be partially available via summary, but access to raw test results or key metadata related to testing methodology may be restricted. These data are often conducted using standardized test methods or guidelines (e.g., OECD, International Organization for Standardization) and represent a reliable source of data. However, concern over protection of confidential business information can discourage sharing. The richness of this data set was enhanced by the sharing of privately owned data, from individual stakeholders and from industry. To alleviate concern over protection of proprietary information while still allowing the shared data to remain useful, creative solutions such as coding to mask data sources and trade names were utilized.

#### Process challenges

Selection criteria structure was critical to returning a fit‐for‐purpose data set. Certain criteria were obvious; for instance, a CAS number was considered crucial to both identify chemicals and link chemical information with test results and classification schemes. However, carefully narrowing other criteria was challenging. To retain the balance between representing a diverse chemical landscape and data useful to the predicted application, criteria such as “effect” or “test statistic” were expanded to include more than simply the standard criteria found in common regulatory test guidance. As an example, broadening test statistic criteria to span a range of percent effect and parameters beyond a common 10% effective concentration allowed the retention of more than 13 000 chronic test result records.

Although most source tables included the same or similar fields of data and metadata, a significant effort was required to harmonize the data so that it could be understood and analyzed as a whole. Challenges were encountered in the harmonization of terminology; for instance, more than 10 different versions of the measured effect designation “growth” were present, some different only in verbiage and others in whether (or how) growth was measured as an increase or decrease. The size of the master data set and the number of original sources created logistical challenges in harmonization. Splitting data and metadata into relationally linked tables, where pieces of data were not typically repeated in all tables (i.e., physico‐chemical information for each CAS stored only in the “compound properties” table) was useful to maximize intratable harmonization to key fields such as CAS and test organism.

#### Critical decisions

A number of decisions were crucial to ensure that the overall data set was shaped to fit the end goal, over and above the determination of stepwise filtering criteria. Most were practical, as in the establishment of which data records and fields were required and their justification for inclusion; one example is the inclusion of test duration, which was necessary to determine whether a test was intended to generate an acute or chronic result—therefore, without it, the data record was unusable for PNEC determination and subsequent ecoTTC and related analyses. Another was the determination of whether a test result was truly considered acute or chronic, which launched the eventual development of an entire decision matrix for acute/chronic classification based on test organism, duration, and other factors. At times, reality drove decision‐making: one example was the impossibility of fully curating the significant mismatching between CAS numbers and SMILES codes given that no single recognized source for CAS or SMILES exists. Improved matching rates for CAS numbers and SMILES codes can be accomplished if scientists realize the great importance of the need to be accurate and improve their understanding of chemistry and a fully curated, publicly available system following expert review is devised.

#### Data heterogeneity

Both test type and species tested have changed over time. For example, the zebrafish is an emergent fish model that has gained favor in the past few years, but much of the historical information is on fathead minnow and bluegill. Algal inhibition (toxicity) testing has changed over time with the evolution of guidelines and guidance (e.g., US Environmental Protection Agency 1996; Organisation for Economic Co‐operation and Development 2011), whereas *Daphnia* toxicity testing protocols have remained relatively consistent (Organisation for Economic Co‐operation and Development 1984, 2008). Species names have also changed over time. Some of the recent changes that impacted the development of EnviroTox include *P. subcapitata* (formerly *Selenastrum capricornutum*), *D. subspicatus* (formerly *Scenedesmus subspicatus*), *D. rerio* (formerly *Brachydanio rerio*), *O. mykiss* (formerly *Salmo gairdneri*), and *Americamysis bahia* (formerly *Mysidopsis bahia*).

The types of endpoints measured have continued to evolve, with an increase in behavioral, genomic, cellular, and other nontraditional assay types. Concurrently, the type(s) and amount of information, and therefore database structure and accessibility, must adapt to capture this new information. This type of data may be included in a future version of the database but was not included in this version of the EnviroTox database.

#### Fundamental issues

The current ecotoxicology data landscape is housed in virtual silos, still largely defined by 2 extremes: 1) large compilations that have not been curated and that have less defined architecture and content, or 2) carefully curated collections of individually sourced or proprietary data that have more limited taxa and chemical diversity because of standardization and data‐selection approaches. In this landscape there has been no middle ground, where pooled data that are well characterized and well understood provide enough breadth and depth to investigate not only single chemicals but trends or patterns across chemical space. The construction of the EnviroTox database moves toward such a data center.

It must be recognized, however, that despite the SIFT data curation that was used to develop the EnviroTox database, the individual records pulled from the accessed databases were not necessarily quality assured by the respective agency or organization. Despite the careful considerations given to the process challenges, the lack of individual record quality assurance beyond what was included in the original database development or via the SIFT EnviroTox process could have resulted in inclusion of some poorer‐quality data.

Another basic barrier to data sharing is the lack of accepted common language within the ecotoxicology community. This not only is true for the most essential terminology (i.e., a test statistic, an endpoint, or an effect measurement) but also extends to descriptions and methodologies. Development of a common language removes the logistical investment in data translation before and after the share. Thinking toward the future of “big” data use in ecotoxicology, this common language prepares the way for development of ontologies, which define relationships that can be used to systematically organize and integrate disparate sources of data on a large scale.

Modern databases can be constructed using efficient database management tools (such as MySQL) and new open‐source coding which more fully utilizes computational power. By using these coding, storage, and information management tools, the likelihood of any given database living well into the future can be maximized.

## CONCLUSIONS

A curated and archived data set has been compiled for the initial purposes of developing ecoTTCs and CTDs as screening tools for chemical hazard assessment. Clearly, such a database can be useful for a wide range of purposes where management of environmental hazard data is required. This new database, EnviroTox, is accessible at http://www.EnviroToxdatabase.org, and it is planned that the database will be updated on a yearly basis. EnviroTox and the underlying database is a public domain platform hosted and maintained by the Health and Environmental Sciences Institute. The database records are stored in a MySQL Environment, and open source R code is used to probe the data. Initial applications are in development and will include deriving PNECs and categorizing mode of action assignments as a means to structure category‐specific evaluations, in addition to the distribution analyses.

## Supplemental Data

The Supplemental Data are available on the Wiley Online Library at DOI: 10.1002/etc.4382.

## Disclaimer

The manuscript was subjected to review by the USEPA's National Health and Environmental Effects Research Laboratory and Environment and Climate Change Canada and approved for publication. Approval does not signify that the contents reflect the views of the USEPA or Environment and Climate Change Canada, nor does mention of trade names or commercial products constitute endorsement or recommendation for use.

## Supporting information

This article includes online‐only Supplemental Data.

Supporting Data S1.

## Data Availability

All information associated with this manuscript is available on the EnviroTox database website at http://www.EnviroToxDatabase.org.
