# Supplementary material for: Creation of a Curated Aquatic Toxicology Database: EnviroTox
Source: Environ Toxicol Chem. 2019 Apr 1;38(5):1062–73. doi: 10.1002/etc.4382 (PMC6850623; doi:10.1002/etc.4382)
Supplement: Supplementary file 1 — Supporting Data S1. [file ETC-38-1062-s001.docx]

**Supplementary Information**

**Creation of a Curated Environmental Toxicology Database: EnviroTox**

Kristin A. Connors^a^, Amy Beasley^b^, Mace Barron^c^, Scott E. Belanger^a^, Mark Bonnell^d^, Jessica L. Brill^a^, Dick de Zwart ^e^, Aude Kienzler^f^, Jesse Krailler^a^, Ryan Otter ^g^, Joshua L. Phillips ^g^, Michelle R. Embry^h*^

^a^The Procter & Gamble Company, Cincinnati, OH USA

^b^The Dow Chemical Company, Midland, MI USA

^c^US Environmental Protection Agency, Gulf Breeze, FL USA

^d^Environment and Climate Change Canada, Gatineau, ON Canada

^e^Mermayde, Groet, The Netherlands

^f^European Commission, Joint Research Centre (JRC), Ispra, Italy

^g^Middle Tennessee State University, Murfreesboro, TN USA

^h^Health and Environmental Sciences Institute, Washington, DC USA

**Table S1.** ECOSAR classifications for the EnviroTox Database & collapsed assignments

| Separated classes - first classification from ECOSAR | Collapsed Assignment |
| --- | --- |
| Acid Halides | Halides |
| Acid moiety | Acids |
| Acrylamides | Acrylamides |
| Acrylates | Acrylates |
| Aldehydes (Mono) | Aldehydes |
| Aldehydes (Poly) | Aldehydes |
| Aliphatic Amines | Aliphatic Amines |
| Alkoxy Silanes | Alkoxy Silanes |
| Amides | Amides |
| Anilines (amino-meta) | Anilines |
| Anilines (amino-ortho) | Anilines |
| Anilines (amino-para) | Anilines |
| Anilines (Hindered) | Anilines |
| Anilines (Unhindered) | Anilines |
| Aziridines | Aziridines |
| Benzodioxoles | Benzodioxoles |
| Benzotriazoles | Benzotriazoles |
| Benzoylcyclohexanedione | Benzoylcyclohexanedione |
| Benzyl Alcohols | Alcohols |
| Benzyl Halides | Halides |
| Benzyl Nitriles | Nitriles |
| Carbamate Esters | Esters |
| Carbamate Esters, Phenyl | Esters |
| Carbonyl Ureas | Ureas |
| Diketones | Ketones |
| Epoxides, mono | Epoxides |
| Epoxides, mono acid subst | Epoxides |
| Epoxides, Poly | Epoxides |
| Esters | Esters |
| Esters (phosphate) | Esters |
| Esters, Dithiophosphates | Esters |
| Esters, Monothiophosphates | Esters |
| Halo Acids | Acids |
| Halo Alcohols | Alcohols |
| Halo Epoxides | Epoxides |
| Halo Ester | Esters |
| Halo Ethers | Ethers |
| Halo Ketones (2 free H) | Ketones |
| Halo Nitriles | Nitriles |
| Haloacetamides | Acetamides |
| Haloimides | Imides |
| Halopyrdines | Pyrdines |
| Hydrazines | Hydrazines |
| Hydroquinones | Hydroquinones |
| Imidazoles | Imidazoles |
| Imides | Imides |
| Inorganic Compound | Inorganic Compound |
| Ketone alcohols | Alcohols |
| Malonitriles | Nitriles |
| Melamines | Melamines |
| Methacrylates | Methacrylates |
| Neutral Organics | Neutral Organics |
| Nitrile Alpha-OH | Nitriles |
| Nitriles, Polyaliphatic | Nitriles |
| Not classified | Not classified |
| Oxime Carbamate Ester | Esters |
| Peroxy Acids | Acids |
| Phenols | Phenols |
| Phenols, Poly | Phenols |
| Phosphine Oxide | Phosphine Oxide |
| Phthalonitriles | Nitriles |
| Polynitrobenzenes | Polynitrobenzenes |
| Propargyl Alcohol-hindered | Alcohols |
| Propargyl Alcohols | Alcohols |
| Propargyl Halide | Halides |
| Pyrazoles/Pyrroles | Pyrazoles/Pyrroles |
| Quinones | Quinones |
| Rosins | Rosins |
| SHOULD NOT BE PROFILED | SHOULD NOT BE PROFILED |
| Substituted Ureas | Ureas |
| Sulfonyl Ureas | Ureas |
| Thiazolones (Iso-) | Thiazolones |
| Thiocarbamate, Di(Substit) | Thiocarbamate |
| Thiocarbamate,Di(Free acid) | Thiocarbamate |
| Thiocarbamates, Mono | Thiocarbamate |
| Thiocyanates | Thiocyanates |
| Thiols and Mercaptans | Thiols and Mercaptans |
| Thiophenes | Thiophenes |
| Thioureas | Ureas |
| Triazines, Aliphatic | Triazines |
| Triazines, Aromatic | Triazines |
| Triazoles (Non-Fused) | Triazines |
| Vinyl/Allyl Alcohols | Alcohols |
| Vinyl/Allyl Aldehydes | Aldehydes |
| Vinyl/Allyl Ethers | Ethers |
| Vinyl/Allyl Halides | Halides |
| Ketone | Ketones |
| Vinyl/Allyl Nitriles | Nitriles |
| Vinyl/Allyl Sulfones | Sulfones |

**Table S2.** Figure 4 details on number of substances and species

| ECOSAR Collapsed  Assignment | Trophic Level | TEST type | chemicals | species |
| --- | --- | --- | --- | --- |
| Neutral Organics | INVERT | A | 443 | 258 |
| Acids | INVERT | A | 78 | 57 |
| Aliphatic Amines | INVERT | A | 189 | 72 |
| Amides | INVERT | A | 79 | 99 |
| Neutral Organics | INVERT | C | 172 | 15 |
| Esters | INVERT | A | 336 | 318 |
| Amides | INVERT | C | 47 | 8 |
| Acids | INVERT | C | 33 | 14 |
| Phenols | INVERT | A | 140 | 283 |
| Anilines | INVERT | A | 106 | 73 |
| Aliphatic Amines | INVERT | C | 70 | 6 |
| Triazines | INVERT | A | 31 | 57 |
| Nitriles | INVERT | C | 19 | 12 |
| Halides | INVERT | A | 35 | 106 |
| Phenols | INVERT | C | 56 | 29 |
| Nitriles | INVERT | A | 28 | 78 |
| Esters | INVERT | C | 131 | 44 |
| Halides | INVERT | C | 16 | 13 |
| Anilines | INVERT | C | 43 | 11 |
| Triazines | INVERT | C | 17 | 11 |
| Neutral Organics | FISH | A | 544 | 165 |
| Aliphatic Amines | FISH | A | 211 | 74 |
| Neutral Organics | FISH | C | 123 | 20 |
| Acids | FISH | A | 92 | 57 |
| Amides | FISH | A | 112 | 93 |
| Anilines | FISH | A | 124 | 47 |
| Esters | FISH | A | 414 | 212 |
| Phenols | FISH | A | 171 | 132 |
| Triazines | FISH | A | 36 | 49 |
| Nitriles | FISH | A | 42 | 75 |
| Aliphatic Amines | FISH | C | 48 | 10 |
| Halides | FISH | A | 50 | 103 |
| Amides | FISH | C | 35 | 10 |
| Acids | FISH | C | 14 | 9 |
| Anilines | FISH | C | 24 | 10 |
| Esters | FISH | C | 103 | 37 |
| Halides | FISH | C | 10 | 9 |
| Phenols | FISH | C | 41 | 27 |
| Triazines | FISH | C | 18 | 11 |
| Nitriles | FISH | C | 16 | 8 |
| Esters | ALGAE | A | 136 | 54 |
| Neutral Organics | ALGAE | A | 220 | 64 |
| Anilines | ALGAE | A | 60 | 22 |
| Acids | ALGAE | A | 50 | 23 |
| Neutral Organics | ALGAE | C | 193 | 31 |
| Amides | ALGAE | A | 68 | 32 |
| Acids | ALGAE | C | 43 | 25 |
| Esters | ALGAE | C | 131 | 39 |
| Amides | ALGAE | C | 63 | 25 |
| Aliphatic Amines | ALGAE | A | 98 | 37 |
| Nitriles | ALGAE | A | 11 | 15 |
| Phenols | ALGAE | A | 86 | 46 |
| Halides | ALGAE | A | 18 | 7 |
| Halides | ALGAE | C | 19 | 4 |
| Aliphatic Amines | ALGAE | C | 68 | 23 |
| Phenols | ALGAE | C | 65 | 27 |
| Nitriles | ALGAE | C | 11 | 13 |
| Triazines | ALGAE | A | 26 | 85 |
| Anilines | ALGAE | C | 51 | 24 |
| Triazines | ALGAE | C | 19 | 43 |


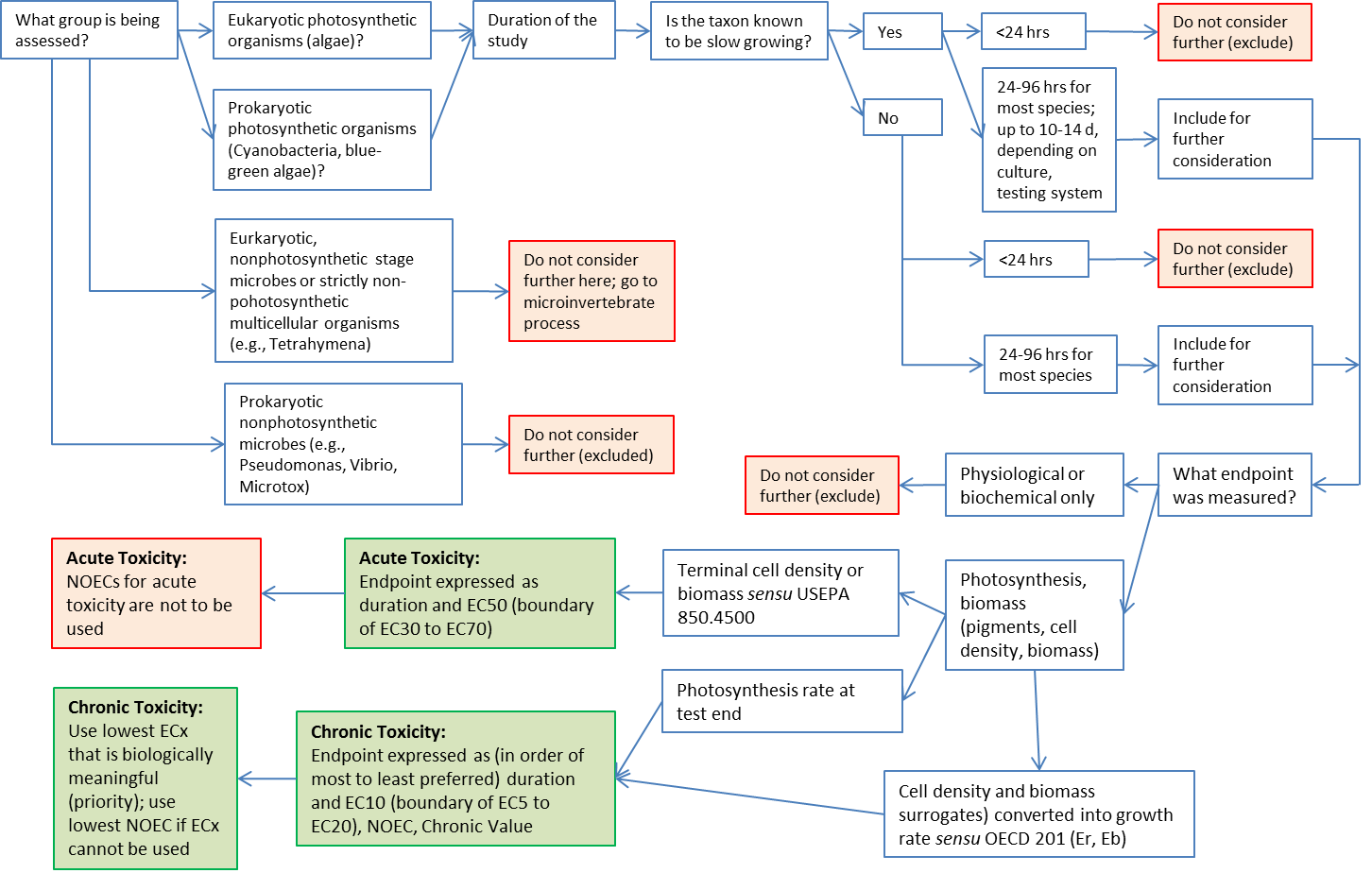


**Figure S1.** Process for classifying photosynthetic micro-organism tests as either acute or chronic in the EnviroTox database.


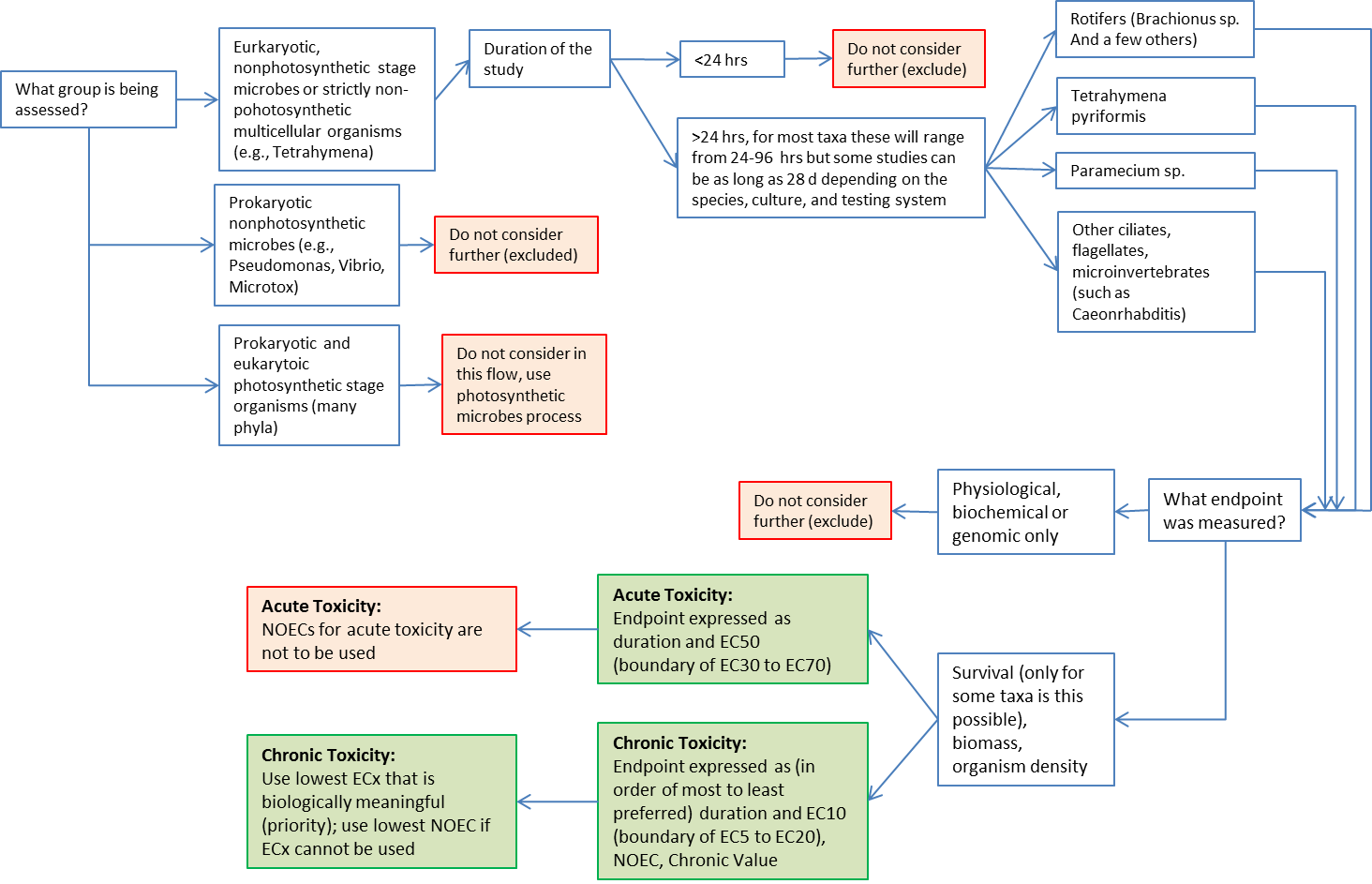


**Figure S2.** Process for classifying non-photosynthetic microbial and microinvertebrate tests as either acute or chronic in the EnviroTox database.


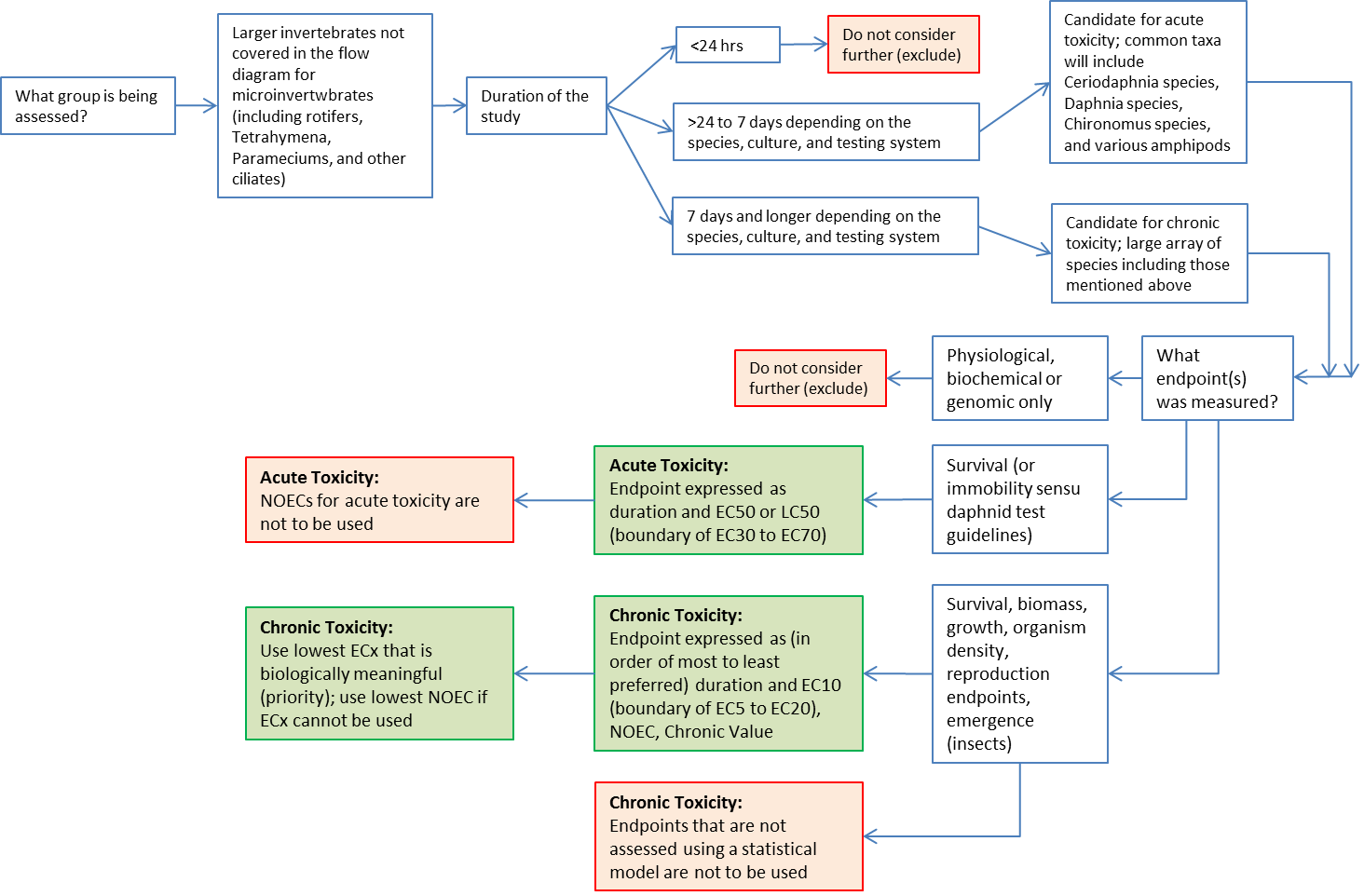


**Figure S3.** Process for classifying macroinvertebrate tests as either acute or chronic in the EnviroTox database.


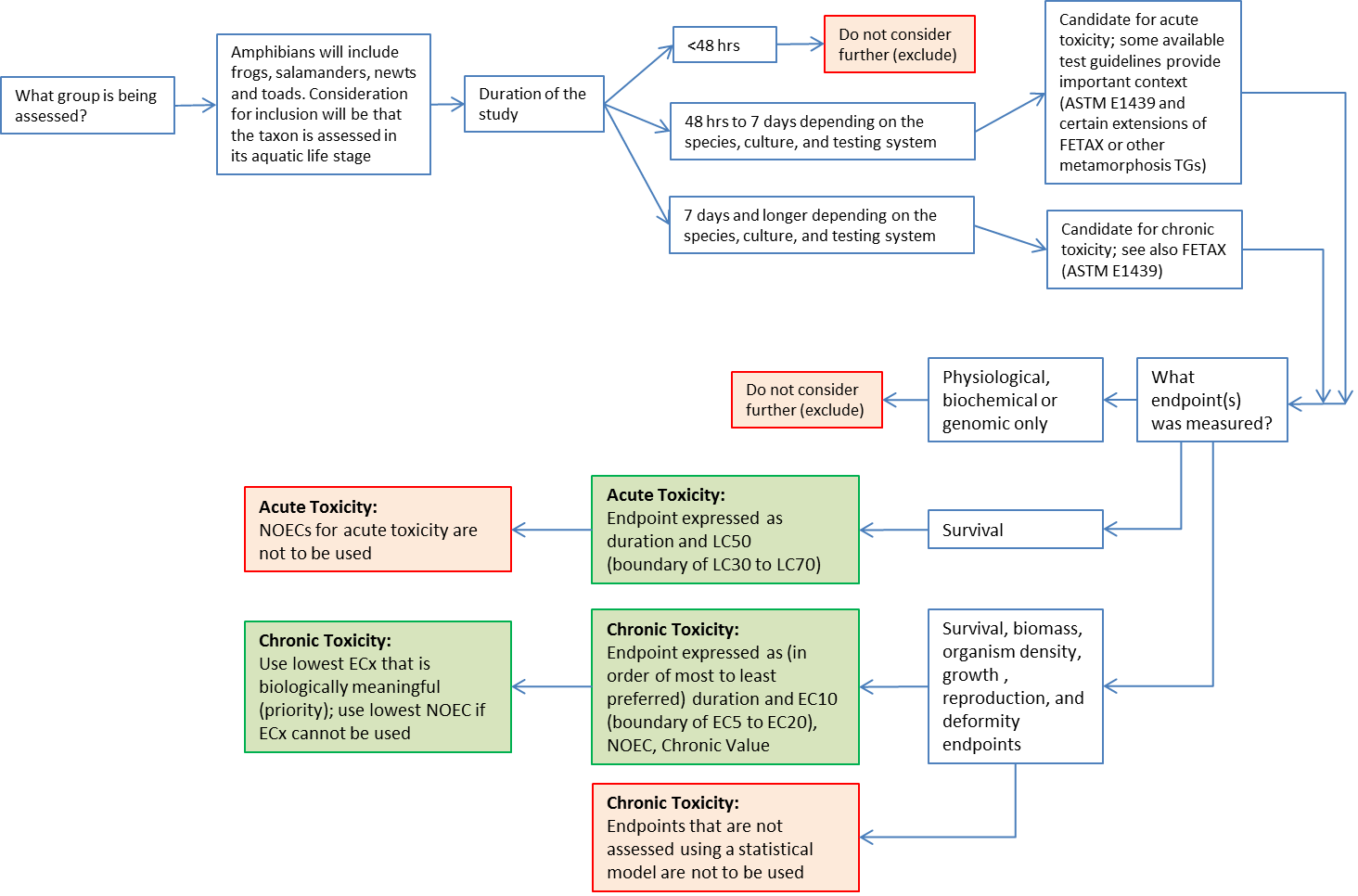


**Figure S4.** Process for classifying amphibian tests as either acute or chronic in the EnviroTox database.


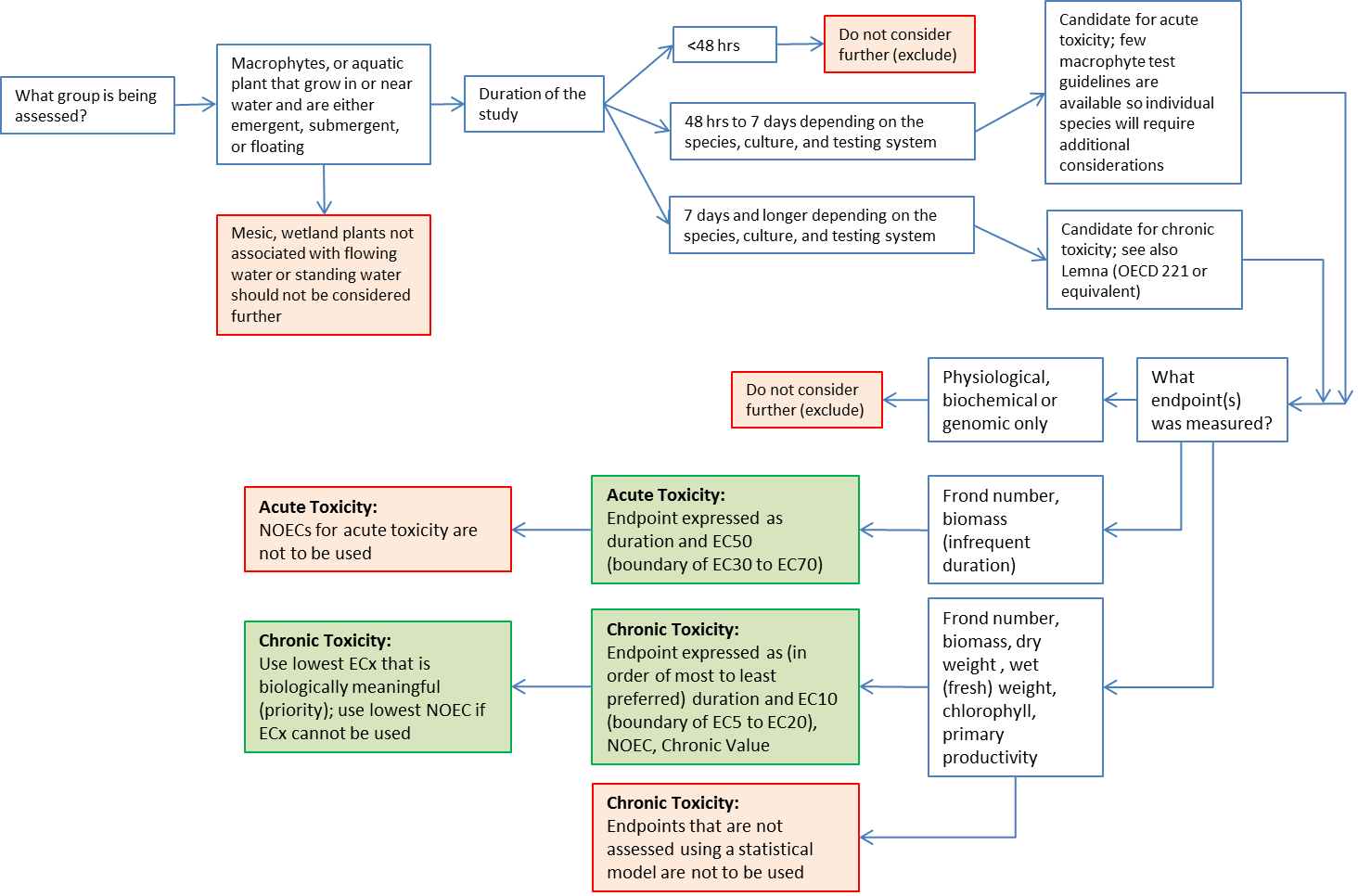


**Figure S5.** Process for classifying macrophyte tests as either acute or chronic in the EnviroTox database.
